# Current and future trends in the consumption, sale and purchasing of alcohol‐free and low‐alcohol products in Great Britain, 2014 to 2023

**DOI:** 10.1111/add.70041

**Published:** 2025-03-21

**Authors:** Luke B. Wilson, Abigail K. Stevely, Inge Kersbergen, Ellen McGrane, Esther C. Moore, Rob E. Pryce, Jamie Brown, John Holmes

**Affiliations:** ^1^ Sheffield Addictions Research Group, School of Medicine and Population Health University of Sheffield Sheffield UK; ^2^ Department of Behavioural Science and Health UCL London UK

**Keywords:** alcohol drinking, consumption, forecasting, purchasing, time series analysis, zero‐alcohol

## Abstract

**Background and Aims:**

The UK Government has committed to reducing alcohol consumption by 2025 through increasing the availability of alcohol‐free and low‐alcohol (no/lo) drinks. This study estimated current and future trends in key indicators of the availability, sale, purchasing and consumption of no/lo products in Great Britain.

**Design:**

Seasonal autoregressive integrated moving average models of market research data and repeat‐cross‐sectional survey data on alcohol consumption.

**Setting:**

Great Britain (England, Scotland and Wales), January 2014–December 2025.

**Participants/Measurements:**

The study used population‐level data on no/lo product availability and sales in the on‐trade (e.g. bars, pubs, clubs, restaurants), as well as the off‐trade (e.g. supermarkets and convenience stores) (2014–2023), continuous household panel data on purchasing (*n* ≈ 30 000; 2018–2023) and repeat‐cross‐sectional survey data on consumption (*n* ≈ 80 000, 2020–2024) to construct monthly time series for seven indicators. It described current trends and forecast them to December 2025.

**Findings:**

All indicators showed increasing trends to 2025. The forecast level of each indicator in December 2025 was: Indicators 1 and 2: Percentage of alcoholic drinks sales volume that is no/lo products: 2.3% (50% Prediction Interval 2.1%–2.9%, off‐trade) and 1.0% (50% Prediction Interval 0.8%–1.1%, on‐trade); Indicator 3: Percentage of pubs selling draught no/lo products: 6.8% (50% Prediction Interval 6.1%–7.5%); Indicator 4: Percentage of households purchasing off‐trade no/lo products but not alcoholic products: 12.3% (50% Prediction Interval 10.9%–13.6%); Indicator 5: Percentage of higher alcohol purchasing households that are increasing off‐trade purchasing of no/lo products: 24.3% (50% Prediction Interval 21.3%–30.6%); Indicator 6: Percentage of households increasing off‐trade purchasing of no/lo products and decreasing purchasing of alcoholic products: 1.8% (50% Prediction Interval 0.8%–2.8%); Indicator 7: Percentage of risky drinkers using no/lo products in most recent cut‐down attempt: 42.4% (50% Prediction Interval 37.2%–53.3%).

**Conclusions:**

Consumption of alcohol‐free and low‐alcohol drinks is increasing in Great Britain but predicted to remain low in 2025 (estimated at 1.0% of on‐trade and 2.3% of off‐trade alcohol sales volume in servings by the end of 2025). There is some evidence that people are using no/lo drinks in attempts to reduce their alcohol consumption.

## INTRODUCTION

Alcohol‐free and low‐alcohol drinks (no/lo drinks) are beers, ciders, wines, spirits and RTDs (ready‐to‐drinks or pre‐mixed spirits, also known as alcopops) containing little or no alcohol. Definitions vary by country, however, the United Kingdom (UK) Government defines alcohol‐free drinks as containing up to 0.05% alcohol by volume (ABV), and low alcoholic drinks as containing more than 0.05% ABV and up to and including 1.2% ABV [1]. These products are an increasingly high‐profile sector of the alcoholic drinks market in high‐income countries [2], with wider availability and choice of products rising in the on‐trade (e.g. bars, pubs, clubs and restaurants), as well as the off‐trade (e.g. supermarkets and convenience stores).

Although news reports make clear the no/lo market has seen rapid growth in the last decade [3], there is little published evidence available on time‐trends in no/lo drinks sales and purchasing. Kokole *et al*. [4] used production and export data to estimate consumption trends for non‐alcoholic beer in the European Union (EU)‐27 countries and the United Kingdom, finding that the volume sold increased across these countries from 0.59 to 1.28 billion litres between 2013 and 2019. This increased the share of beer production volumes accounted for by non‐alcoholic beer from 1.8% to 3.8%. A study of supermarket loyalty card data from Finland also found non‐alcoholic beer purchases increased from 2.3% to 3.7% between 2017 and 2018. We are unaware of more recent data in the published literature or of projections of future trends.

If recent growth continues or accelerates, no/lo products could have a transformative impact on public health if people drink no/lo products instead of standard alcoholic drinks. Evidence has shown that reducing the average strength of alcoholic drinks by 10% in the United Kingdom in 2019 would lead to an 8.1% reduction in alcohol attributable deaths in women and a 6.1% reduction in men [5]. However, public health actors have expressed concerns about potential risks if commercial actors market no/lo drinks as additions to existing alcohol consumption, seek to normalise alcohol branding or consumption in new settings (e.g. at work, in the morning, at gyms), use no/lo drinks to subvert alcohol marketing restrictions (e.g. bans on billboard advertising) or leverage any success of no/lo drinks to secure greater influence over wider alcohol policy [2].

In contrast, industry actors present no/lo drinks as a solution to alcohol harm and some include objectives to increase no/lo sales in their corporate social responsibility strategies. For example, the multinational brewer AB InBev's Global Smart Drinking Goals include ensuring no alcohol and lower strength beers (up to 3.5% ABV) comprise at least 20% of their global sales volume by the end of 2025 [6]. However, evaluations of previous industry pledges related to public health have been criticised for setting targets that are in line with pre‐existing trends, which would likely have been reached without any further intervention or relate to measures that are unlikely to improve public health [7, 8].

Nonetheless, the UK Government's green paper, ‘*Advancing our health: prevention in the 2020s*’, committed to working with alcohol producers and retailers to increase substitution of alcoholic drinks with no/lo alcohol alternatives among people who drink above low risk levels and deliver a significant increase in the availability of no/lo drinks in the on‐ and off‐trade by 2025 [9]. To support this aim, the government requires evidence to inform decision‐making, implementation and evaluation of the policy.

This study aimed to: (1) characterise trends for seven key indicators of no/lo sales, purchasing and consumption in Great Britain between 2014 and 2023, with the time period differing across indicators because of data availability; and (2) forecast future trends in these indicators to the end of 2025 to inform target‐setting for the end‐date of the current UK Government policy. This study used data from four sources, each providing a different insight into the consumption, sales and purchasing of no/lo beverages. All have been used in previous evaluations of UK alcohol policy [10, 11, 12, 13].

## METHODS

### Data

#### On‐trade sales data

Weekly on‐trade alcohol sales data was purchased from *CGA* by NielsenIQ (hereafter CGA) for 499 weeks from June 2014 to December 2023. The CGA data comprise weekly sales data in Great Britain by natural volume (i.e. mL of product) and value for individual named stock‐keeping units (SKUs) (e.g. 12 × 330 mL Heineken 0.0 or 700 mL Gordons 0.0) as well as additional product characteristics (e.g. ABV, container size). CGA estimates alcohol sales using a combination of electronic point of sales (EPOS), delivery and survey data collected from a stratified sample of on‐trade retailers. The data was provided across five drink categories: beer, cider, wine, spirits and RTDs for both standard alcoholic and no/lo products (although data for standard alcoholic products is aggregated to the drink category level rather than at the SKU level).

#### Off‐trade sales data

Weekly off‐trade alcohol sales data was supplied by Circana (formerly IRI) for 208 weeks from January 2020 to December 2023. Circana provided SKU‐level sales data by natural volume and value of weekly sales as well as product characteristics (e.g. ABV, container size). Circana estimates alcohol sales in Great Britain using a combination of EPOS and wholesale data. EPOS data is collected from a census of large multiple retailers (supermarkets, excluding discount stores such as Aldi and Lidl) and a stratified sample of smaller convenience retailers. Where Circana is unable to obtain census data, EPOS data is collected from a sample of retailers and modelled to represent the retailer group. The data was provided across seven drink categories: beer, cider, perry, wine, fortified wine, spirits and flavoured alcoholic beverages (FABs) (equivalent to RTDs in the CGA data, referred to as RTDs hereafter) in both standard alcoholic and no/lo beverage types. For the purposes of this study, we grouped cider and perry together to match the beverage categories in CGA.

#### Household‐level off‐trade purchasing data

Kantar's WorldPanel (KWP) is a longitudinal household‐level panel study that provides data on off‐trade alcoholic and no/lo purchases from all store types. We purchased KWP data from January 2018 to December 2023, including the natural volume of the product, SKU, packaging type (bottles/cans *etc*.), number of items in any multipacks and the ABV. The KWP sample consists of approximately 30 000 households in Great Britain at any one time, recruited through stratified quota sampling, with quotas set for geographical region, household size, age of main shopper and occupational social grade. The same households provide longitudinal data over time, with continuous replenishment to replace households that leave the sample and ensure the panel remains representative of households in Great Britain. Households record all off‐trade purchases from all store types that are brought back into the home, including purchases made on‐line, using barcode scanners provided by Kantar. To be included in the dataset, households must meet quality control criteria, including meeting thresholds for data recording and purchasing volume and spend (based on household size) every 4 weeks. Panellists also upload digital images of shopping receipts, which KWP uses to verify the accuracy of the household's scanner data. KWP includes survey weights that we use in all analyses.

The Circana, CGA and KWP datasets contain no identifiable missing data as they are compiled automatically from EPOS systems and scanned barcodes. This makes it impossible to distinguish between sales/purchases that did not take place and those that were not recorded.

#### Individual‐level consumption data

The Alcohol Toolkit Study (ATS) is a monthly survey that collects individual‐level data on standard alcohol consumption including the Alcohol Use Disorders Identification Test‐Consumption (AUDIT‐C). The AUDIT‐C is a validated screening test for harmful drinking [14]. The ATS asks hazardous or harmful drinkers [defined as those with an AUDIT‐C ≥8 (harmful) or AUDIT‐C ≥5 (hazardous)] who tried to cut down at least once in the past year, which method(s) they used to cut down, including ‘Low‐alcohol and alcohol‐free drinks’. Data on using no/lo products to cut down on standard alcohol was first collected in October 2020 among the Great Britain sample and the most recent wave of data at the time of analysis was from January 2024. The ATS is a nationally representative telephone survey among people aged 16 and over who live in private households in Great Britain (*n* = ~1700 in England, *n* = ~450 in Scotland and *n* = ~300 in Wales each month). We included all ages in the analyses, including 16 to 17 year‐olds. The study uses a mixture of random probability and simple quota sampling. Data is collected via computer assisted telephone interviews. The ATS dataset includes survey weights that we use in all analyses. Detailed methods are described in the published protocol and on‐line (http://www.alcoholinengland.info) [15]. Additional descriptive statistics on the ATS can be found in the Supporting information Section 8.

### Coronavirus disease 2019

The coronavirus disease 2019 (Covid‐19) pandemic occurred during our study period and changed behaviours surrounding alcohol and alcohol consumption, such as drinking location, drinking days per week and drinking start times [16]. Governments implemented ‘lockdown’ measures to reduce social contact and the spread of the virus. This led to the temporary closure of all on‐trade venues in the United Kingdom, meaning there were effectively no on‐trade alcohol sales during this period. These closures were followed by incremental reopening under restrictions that limited venue capacity and, therefore, trading hours and sales.

To control for the Covid‐19 pandemic we include the Oxford Covid‐19 Government Response Tracker (OxCGRT) for the United Kingdom as a covariate in all analyses [17]. The OxCGRT provides indexed values indicating the severity of societal restrictions implemented by governments worldwide to hinder Covid‐19 transmission. Previous research suggests the OxCGRT controls effectively for the impact of the pandemic on alcohol‐related outcomes [18].

### Calculating the servings of no/lo drinks

For several of the indicators described below, we compare no/lo sales volume with standard alcohol sales. Public health‐oriented analyses of alcohol sales volumes typically convert volume of liquid into volume of ethanol in grams, standard drinks or units. However, this would be uninformative for no/lo drinks as they contain little or no alcohol. Using volume of liquid would also be problematic because people switching from standard alcoholic drinks to no/lo spirits would have a smaller impact on the volume of no/lo liquid sold than if they switched to no/lo beer because beer is sold in larger serving sizes. However, the reduction in alcohol consumption may be the same in both scenarios.

We address this problem by using ‘servings’ as a volume metric rather than natural volume. To calculate servings, we divide the natural volume of sales by a standard serving size for each beverage type. We, then, sum together servings for all beverage types to give the total sales volume in servings. To select the standard serving sizes used for the on‐trade, we used data from the Weights and Measures Act 1985 (https://www.gov.uk/weights-measures-and-packaging-the-law/specified-quantities), which specifies that alcoholic beverages must be sold in fixed sizes known as ‘specified quantities’. To select the standard serving sizes used for the off‐trade, we calculated the median product volume by SKU for each beverage type in Circana, excluding volumes above 1 L to prevent multi‐packs of beer from skewing the results. Because wine and spirits are sold in bottles, for these beverage types we draw on estimates from experimental studies that suggest a self‐poured glass of wine is typically around the size of a medium glass (i.e. 175 mL) and a self‐poured measure of spirits is typically around the size of a double serving (i.e. 50 mL) [19]. Table 1 provides a breakdown of our serving size assumptions for the on‐ and off‐trade. See Supporting Information Section 2 for further discussion of this issue.

**TABLE 1:** Serving size assumptions for on‐ and off‐trade alcoholic beverages.

	On‐trade	Off‐trade
Beer	568 mL	330 mL
Cider	568 mL	500 mL
Wine	175 mL	175 mL
Spirits	25 mL	50 mL
RTDs	250 mL	250 mL

Abbreviation: RTDs, ready‐to‐drinks or pre‐mixed spirits.

### Indicators of no/lo sales, purchasing and consumption

We use seven indicators of no/lo sales, purchasing and consumption, which are calculated from our four datasets and are, therefore, available for different time periods (see Supporting information, Appendix A1 for dataset, time period and the frequency and number of data points). Indicators using sales and purchasing data may be affected by stockpiling, where consumers buy products and keep them for some time before consumption. We selected these indicators in consultation with the UK Department of Health and Social Care as the current analyses were designed to inform their policy development and monitoring. Our indicators are as follows:

1. Percentage of total on‐trade alcohol sales volume that is no/lo: the numerator and denominator are both expressed in servings (see above).
2. Percentage of total off‐trade alcohol sales volume that is no/lo: the numerator and denominator are both expressed in servings (see above).
3. Percentage of on‐trade outlets offering a no/lo beer on draught: draught beer is dispensed into glass from a tap on the bar, as opposed to sold in a bottle or can, and is, therefore, more visible to the consumer. The denominator for the percentage is the total number of licensed outlets. Increasing the availability of draught no/lo beer is attractive to policy‐makers because it makes these products more visible and normalised. It may also improve perceived value for money by increasing the serving size of no/lo beers and improve perceived quality where consumers prefer draught beer to packaged alternatives.
4. Percentage of households who do not normally purchase alcohol that are purchasing no/lo products (off‐trade only): calculated in KWP for each week based on whether households in the sample in that week have purchased no/lo and alcoholic drinks in the preceding year. Households not present continuously in the survey for at least 12 months were excluded from analyses using this measure as they could not be robustly identified as non‐alcohol purchasers in the last year.
5. Percentage of increasing or higher risk households who are increasing their purchasing of no/lo products relative to alcohol products (off‐trade only): increasing or higher risk households are those that purchased more than 14 units (1 UK unit = 8 g/10 mL ethanol) of alcohol per adult in the household per week on average over the previous 12 months [20]. For each increasing or higher risk household in KWP each week, we calculated the number of no/lo servings and standard alcohol servings purchased during the previous 52 weeks. We, then, calculated no/lo servings and standard alcohol servings purchased during the current week and previous 51 weeks, and identified whether they purchased a greater proportion of no/lo servings compared to standard alcohol servings in this more recent period. We, then, calculated the percentage of households whose no/lo purchasing increased relative to standard alcohol. As above, households not present continuously in the survey for at least 12 months and 1 week consecutively were excluded from analyses using this measure.
6. Percentage of households who are increasing their purchasing of no/lo products and decreasing their purchasing of alcohol products: this is similar to indicators 4 and 5 except indicator 6 includes all alcohol‐purchasing households and, instead of calculating a ratio, identifying households where there was an increase in purchased servings of no/lo drinks and a decrease in servings of standard alcoholic drinks.
7. Percentage of hazardous or harmful drinkers who are trying to cut down their alcohol consumption that used no/lo products in their most recent attempt: the ATS did not include no/lo products as a method of cutting down in England in five waves (May 2022, July 2022, September 2022, November 2022, December 2022, July 2023), but did include it in Wales and Scotland. We, therefore, used multiple imputations to estimate data for England in these waves (see Supporting information, Appendix Section 3 for details). We calculated a monthly time series for the percentage of respondents per wave who used no/lo in their most recent attempt to cut down. The analysis was not pre‐registered, and therefore, the results should be considered exploratory.

### Analysis

We conducted time series analyses using seasonal autoregressive integrated moving average (SARIMA) models. The SARIMA analysis had three stages: (1) fitting a SARIMA model to the observed time series for the period where data is available; (2) using this model to forecast the series values until the end of 2025; and (3) computing prediction intervals and validating the model.

The SARIMA analyses model the trend in the indicator, adjusted using terms for autocorrelation and seasonality, and controlling for the impact of the Covid‐19 pandemic using time series data on changing levels of restrictions from the OxCGRT series. During the model fitting process, we identified candidate models by examining autocorrelation and partial autocorrelation plots and adjusted these models based on correlograms of the errors. If the indicator series showed a strong and consistent seasonal pattern, we tested seasonal differencing. Otherwise, seasonal patterns are modelled using seasonal autoregressive (AR) or moving average (MA) terms. To inform our model selection for each indicator we used the Bayesian information criterion (BIC) to select the most parsimonious model and performed portmanteau tests to confirm that model residuals resembled a white noise process. Where we were unable to fit a model with white noise residuals, we considered using log transformation, integration or aggregating the series to monthly or quarterly time points and tested these models using the augmented Dickey‐Fuller test. For indicator 1, we used monthly rather than weekly time points.

Using the final model specification fit to all the available data, we produced dynamic multistep *ex‐ante* (before the event) forecasts until the end of 2025, the end‐date for the UK Government's commitment to increase the availability and consumption of no/lo drinks. For each week/month of the forecast, we computed 25%, 50% and 95% prediction intervals to describe uncertainty around the estimates. We obtained the size of the prediction interval by estimating the standard deviation of the h‐step forecast distribution and multiplied its square root by the desired coverage probability assuming normally distributed forecast errors to obtain the size of the interval at each value of h (forecast point). Model fit, specifications and validation are found in Supporting information, Appendix Table A2 and A3.

All analyses were conducted using Stata/MP4 version 18 software (StataCorp, College Station, TX).

## RESULTS

### Indicator 1: Percentage of total alcohol sales volume that are no/lo in the on‐trade

**FIGURE 1:**
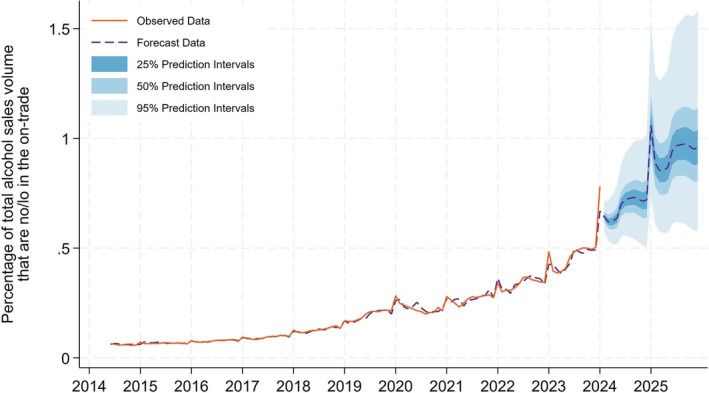
Percentage of total alcohol sales volume that are no/lo in the on‐trade.

The percentage of total alcohol sales volume that are no/lo in the on‐trade has been increasing at a steadily accelerating rate since 2016 (Figure 1). At the end of our sample period (December 2023) the percentage of total alcohol sales volume in servings that are no/lo in the on‐trade is 0.8% If trends continue as they currently are, by the end of 2025 this is expected to rise to 0.9% (50% prediction interval = 0.8%–1.1%). The prediction intervals presented in this time series are asymmetric due to this series being log transformed for analysis.

### Indicator 2: Percentage of total alcohol sales volume that are no/lo in the off‐trade

**FIGURE 2:**
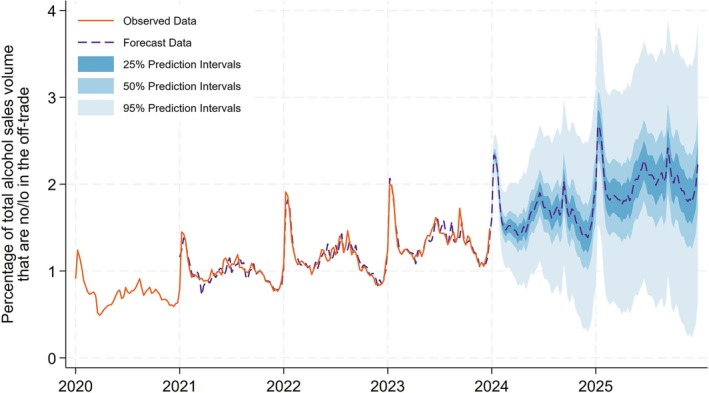
Percentage of total alcohol sales volume that are no/lo in the off‐trade.

The observed data shows substantially greater seasonality than the on‐trade data, with a large peak in January and smaller peaks in the summer months (Figure 2). The trend increases steadily to reach 1.5% by the end of December 2023. The forecast model estimated that this will rise to 2.3% by the end of 2025 (50% prediction interval = 2.1%–2.9%). The shorter time series for the Circana data means the prediction intervals are wider than our on‐trade market forecasts.

### Indicator 3: Percentage of on‐trade outlets offering a no/lo beer on draught

**FIGURE 3:**
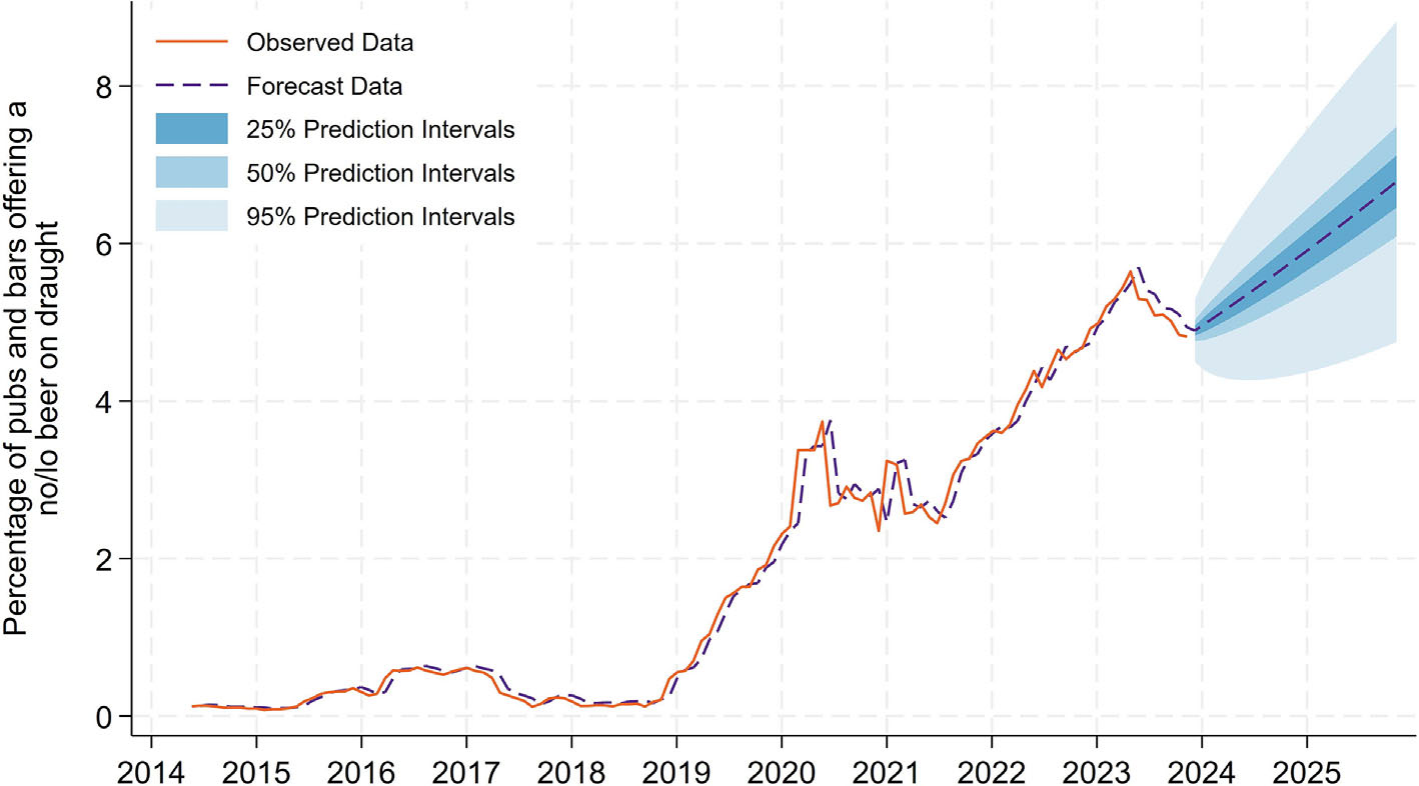
Percentage of pubs and bars offering a no/lo beer on draught.

Figure 3 presents the percentage of on‐trade outlets offering a no/lo beer on draught. In the observed data, we find that the availability of no/lo beers on draught remains largely flat until the middle of 2018 and then starts to increase rapidly. There is a fluctuating trend in draught no/lo beers in on‐trade outlets during the Covid‐19 pandemic when the on‐trade was fully or partially closed, meaning the denominator contains different venues at different times. The upward trend then resumes at the start of 2021 and reaches 4.8% of on‐trade outlets at the end of our observed data period (December 2023), although this is down from a peak earlier in 2023 of 5.6%. In our forecast model we find that, if current trends continue, the percentage of on‐trade outlets offering a no/lo beer on draught will be 6.8% (50% prediction interval = 6.1%–7.5%) at the end of 2025.

### Indicator 4: Percentage of households who do not normally purchase alcohol that are purchasing no/lo products

**FIGURE 4:**
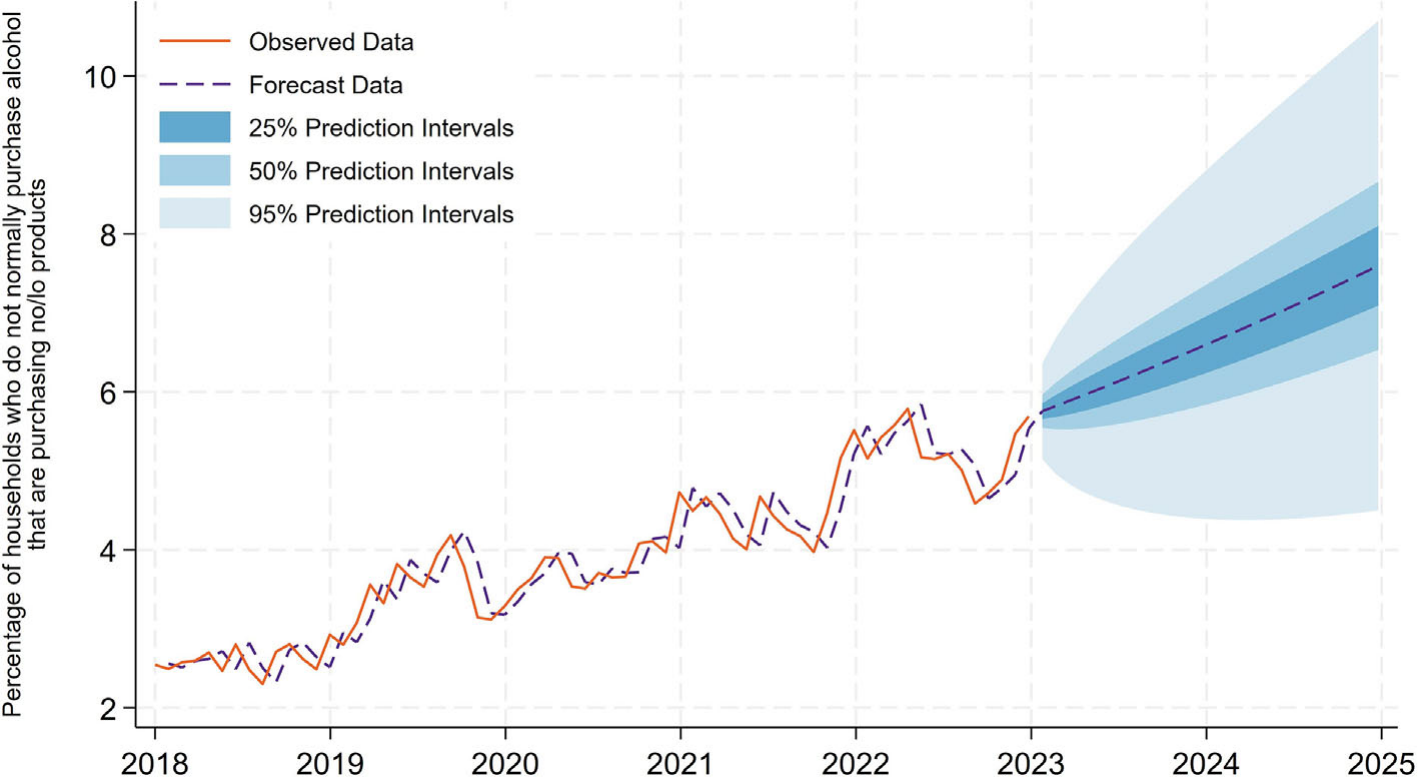
Percentage of households who do not normally purchase alcohol that are purchasing no/lo products.

There has been a steady increase in the percentage of households who do not usually purchase alcohol that are purchasing no/lo products (Figure 4). There was a slight drop in 2019, however, this trend recovers by the end of our observed data. At the end of our observed data 5.7% of non‐alcohol purchasing households now buy no/lo products. We forecast that by the end of 2025 this upward trend continues to 7.6% (50% prediction interval = 6.5%–8.7%).

### Indicator 5: Percentage of increasing or high risk households who are increasing their purchasing of no/lo products (relative to alcohol products) in the off‐trade

**FIGURE 5:**
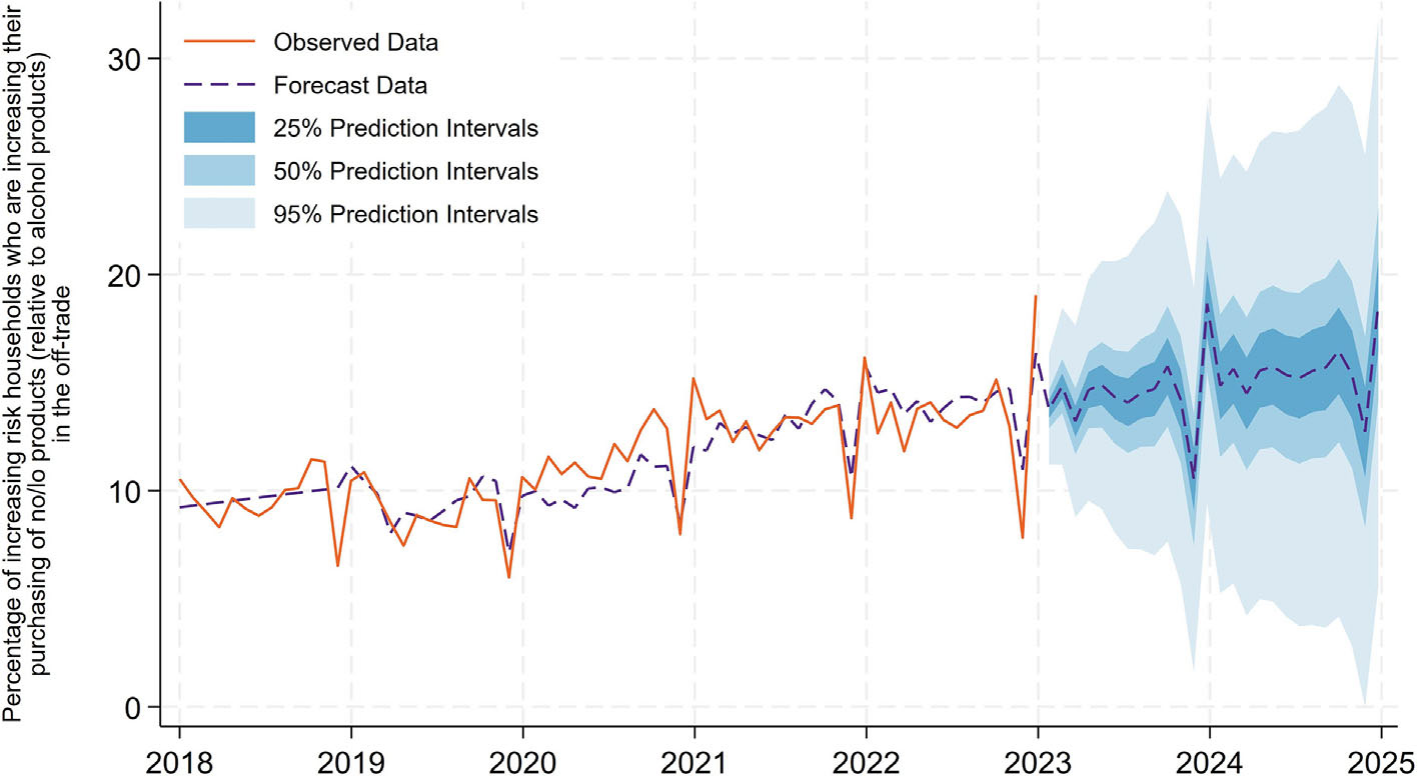
Percentage of increasing or high risk households who are increasing their purchasing of no/lo products (relative to alcohol products) in the off‐trade.

In Figure 5, we find that there are seasonal drops in the percentage of increasing risk households who are increasing their purchasing of no/lo products around the winter holiday period. This drop recovers during the start of the following year. We find at our last period of observed data in December 2023, 19.1% of increasing risk households are increasing their purchasing of no/lo products (relative to alcohol products) in the off‐trade. We estimate that this would fall slightly to 18.6% in December 2025 (50% prediction interval = 14.1%–23.1%).

### Indicator 6: Percentage of households who are increasing their purchasing of no/lo products and decreasing their purchasing of alcohol products

**FIGURE 6:**
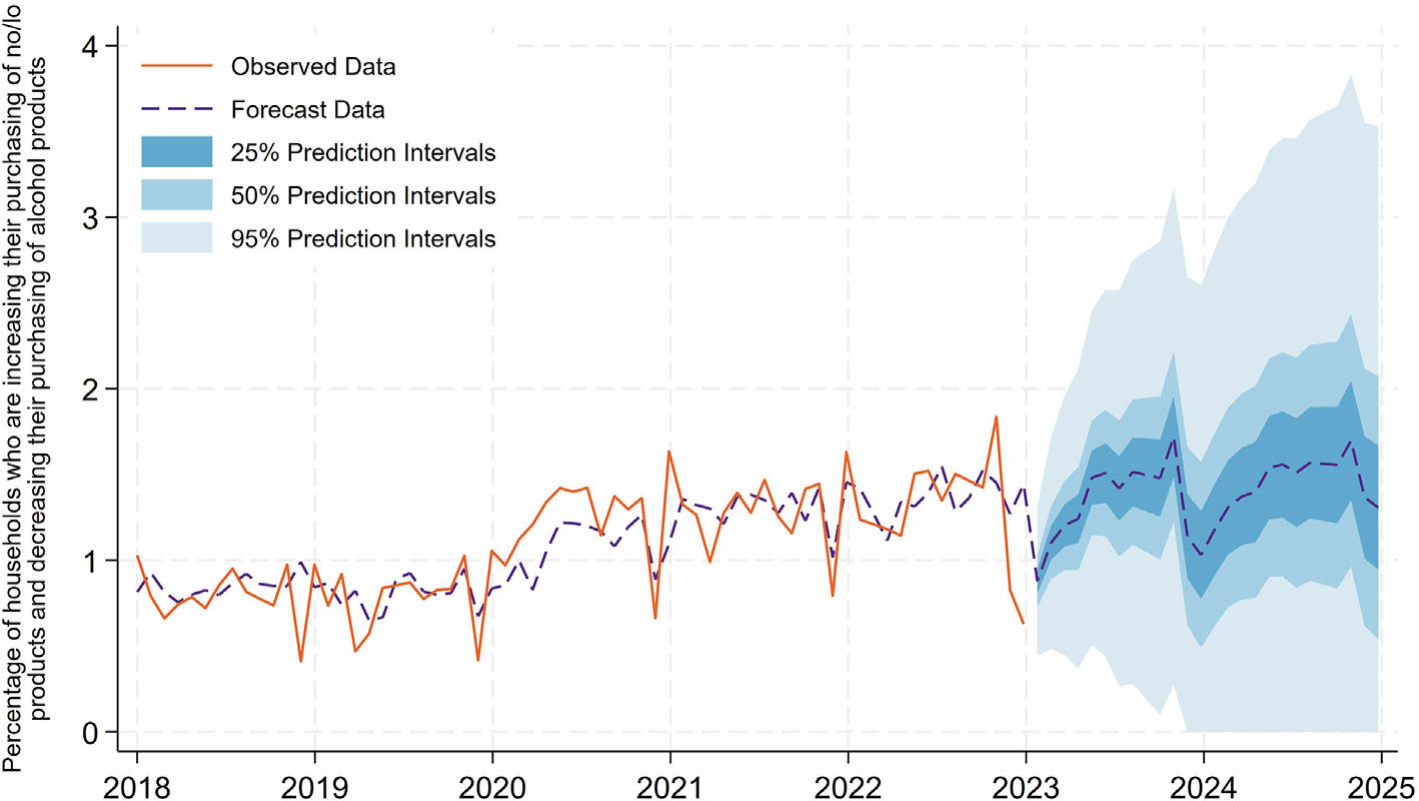
Percentage of households who are increasing their purchasing of no/lo products and decreasing their purchasing of alcohol products.

In Figure 6, we present the percentage of households who are increasing their purchasing of no/lo products and decreasing their purchasing of alcohol products. Like Figure 5, we find that although there are seasonal fluctuations in this indicator, particularly during the winter holiday period there is a small upward trend. In our last period of observed data, we find that the percentage of households who are increasing their purchasing of no/lo products and decreasing their purchasing of alcohol products is 0.6% in December 2023 following a sharp seasonality drop. However, we predict that this would have recovered to 1.3% (50% prediction interval = 0.9%–2.1%) in December 2025.

### Indicator 7: Percentage of hazardous or harmful drinkers self‐reporting using no/lo products in their most recent attempt to cut down

**FIGURE 7:**
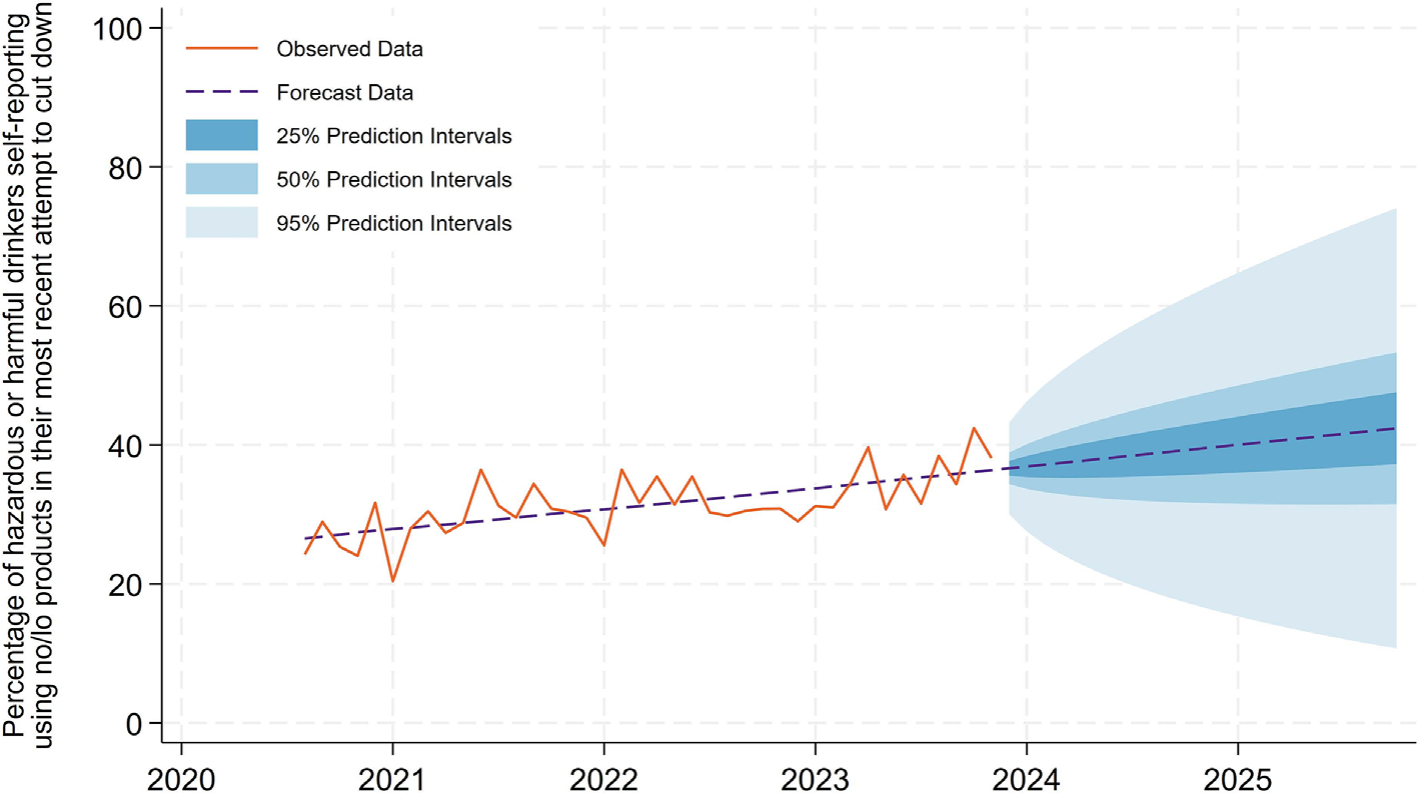
Percentage of hazardous or harmful drinkers self‐reporting using no/lo products in their most recent attempt to cut down.

Figure 7 shows the percentage of hazardous or harmful drinkers self‐reporting using no/lo products in their most recent attempt to cut down. The percentage fluctuates over time, but there is an overall increasing trend. In October 2020, the percentage of hazardous or harmful drinkers self‐reporting using no/lo products in their most recent attempt to cut down was 24.6% rising to 38.1% in our last wave of observed data in December 2023. By the end of 2025, we forecast the percentage of hazardous or harmful drinkers self‐reporting using no/lo products in their most recent attempt to cut down to rise to 42.4% (50% prediction interval = 37.2%–53.3%) in December 2025. All model coefficients can be found in the Supporting Information Section 7.

## DISCUSSION

This study used four datasets to examine how trends in key indicators of no/lo drinks sales, purchasing and consumption have developed in Great Britain between 2014 and 2023. It also modelled and forecast these trends to estimate what these outcomes may look like if current trends continue to the end of 2025, the end‐date for the UK Government's current no/lo policy. Overall, the results suggest that sales, purchasing and consumption of no/lo drinks have expanded substantially from a low baseline and that they will continue to do so to the end of 2025. However, on current trends they will remain only a small contributor to the alcohol market, with no/lo products estimated to account for 1.0% of on‐trade and 2.3% of off‐trade alcohol sales volume in servings by the end of 2025. Alongside this, the results suggest no/lo products are playing a large and increasing role in attempts by heavier drinkers trying to reduce their alcohol consumption, although the present analysis provides no evidence on whether these products are either causing more attempts to cut down or making those attempts more successful.

Evidence that more people are using no/lo drinks when attempting to reduce their alcohol consumption is in line with previous research on no/lo purchasing trends that found launching new lower‐strength beers may reduce overall alcohol purchasing [21], and that increasing the volume of lower‐strength beer in household purchases is associated with a reduced number of units of alcohol within the average shopping basket (but not necessarily reduced overall alcohol purchasing by households) [22, 23]. This evidence, however, primarily examines lower‐strength beers up to 3.5% ABV rather than no/lo drinks below 1.2% ABV. Further qualitative research has also found that people attempting to reduce their alcohol consumption or maintain abstinence describe no/lo drinks as a strategic tool that facilitates moderation and social inclusion [24, 25]. Therefore, despite their relatively small contribution to the alcohol market, there remain good indicators, but insufficient clear evidence, that no/lo drinks may be contributing to reductions in alcohol‐related harm.

This is the most detailed study to date on the current and future trends in the sales, purchasing and consumption of alcohol‐free and low‐alcohol products. It is the first to use sales data on no/lo drinks the gold‐standard data source in privatised alcohol retail markets and uses the high frequency of this data to estimate robust time series models to characterise and forecast trends. We also used wider datasets to include measures not previously examined that capture different aspects of changing consumer behaviours, particularly among hazardous or harmful drinkers. These measures provide novel insights into how individuals are using no/lo products.

However, there are some limitations to note. Our forecast results assume that trends in our indictors will continue on the same trajectory that is seen in the observed period. If there are any policy or market changes regarding no/lo drinks, including major new marketing campaigns or product launches, or if the diffusion of no/lo into the market naturally slows as no/lo consumption becomes more widely adopted, then these will not be accounted for. Our results are models and forecasts, therefore, cannot provide causal evidence regarding any role for no/lo products in reducing alcohol consumption. In addition, although each indicator on their own is limited, when you consider them together you get a greater understanding of the no/lo market and no/lo drinking behaviours. KWP and ATS data rely on self‐reported measures. In general, population surveys produce estimates of population‐level alcohol consumption that are substantially lower than objective measures based on sales or tax data [26], although no study to date has examined the accuracy of reporting of no/lo drink consumption. Because the ATS is cross‐sectional, past hazardous or harmful drinkers who have reduced consumption to the extent that they were classified as low risk drinkers on the AUDIT‐C were not asked to report on cut down attempts in the past year. There is no method available to identify these respondents. However, the ATS is an established data source and has been used in many previous evaluation studies [27, 28], whereas the KWP uses a range of techniques, including photographs of receipts and minimum reporting standards, to ensure the accuracy of reports.

Future research in this area should explore further the relationship between no/lo and standard alcoholic drink sales and consumption. This is a critical question for determining the public health impact of no/lo drinks. Stakeholders particularly require evidence that allows for stronger inferences on the causal relationship between consumption of no/lo and standard alcoholic drinks. The evidence presented in this article showed that all indicators had increasing trends to 2025. However, the analyses provide no evidence on potential health inequalities. For example, it is not known is how these indicators vary by socio‐economic status. Previous research found that no/lo use is less prevalent in less advantaged groups [29]. However, evidence in this area remains sparse and it is unclear whether this is a lagged effect or whether this socio‐economic difference will persist or grow. There is also no quantitative or qualitative evidence on how no/lo drinks have been used by heavier drinkers from less advantaged backgrounds. Analyses of contemporary individual‐ or household‐level panel data and evaluation of largescale interventions or further natural experiments, alongside further qualitative research targeting disadvantaged groups would all be beneficial in this area.

Our results show that the alcohol‐free and low‐alcohol market is continuing to grow, but remains at a level that is unlikely to lead to substantial public health impact by 2025. Additional action to stimulate this market may, therefore, be required if the government continues to view the promotion of no/lo drinks as a key component of its alcohol policies. Although our evidence has shown that higher risk households are increasing their purchasing of no/lo products and risky drinkers are using no/lo drinks in their most recent attempt to cut down on their drinking, less is known on the longevity of these measures, and whether no/lo drinks have contributed causally to any reduction in their alcohol consumption, as opposed to simply replacing other non‐alcoholic beverages or non‐drinking in cut down attempts that would have happened anyway. In 2022, there were 10 048 deaths from alcohol‐specific causes registered in the United Kingdom, the highest number on record. This was 4.2% higher than in 2021 and 32.8% higher than in 2019 [30]. A rise that has been seen in many other countries [31]. Some public health actors have expressed concerns that a focus on no/lo drinks by government may distract attention from adopting more evidence‐based alcohol policy options, such as increasing alcohol prices through taxes or price floors, reducing alcohol availability and restriction on alcohol marketing. Our findings suggest that those concerns may be warranted, although this may change if the no/lo market growth continues or accelerates and leads to clear evidence of substitution.

Although the no/lo market remains only a small percentage of the total alcohol market, the no/lo market will likely continue to expand. This study adds to the growing evidence base on the no/lo drinks market in Great Britain and drinking behaviours.

## AUTHOR CONTRIBUTIONS

All authors had access had to the data used in this study. **Luke B. Wilson** and **Abigail K. Stevely** accessed and verified the underlying data. **Luke B. Wilson** and **Abigail K. Stevely:** Formal analysis (lead); data curation (lead); methodology (lead); software (lead). **Luke B. Wilson:** Writing—original draft (lead); visualisation (lead); validation. **Abigail K. Stevely:** Validation (lead); writing—review and editing. **Robert Pryce, Esther C. Moore**, **Ellen McGrane** and **Inge Kersbergen:** Data curation; formal analysis; writing—review and editing. **Jamie Brown:** Funding acquisition; writing—review and editing. **John Holmes:** Supervision; Funding acquisition; writing—review and editing.

## DECLARATION OF INTERESTS

None.

## Supporting information


**Data S1.** Supporting Information

## Data Availability

Data will not be shared. Data used in these analyses are a commercial product licensed for use by the University of Sheffield and cannot be shared. The data are available for in‐person inspection at the University of Sheffield by researchers on request. Analytical code can be shared on request.
