# Supplementary material for: Current and future trends in the consumption, sale and purchasing of alcohol‐free and low‐alcohol products in Great Britain, 2014 to 2023
Source: Addiction. 2025 Mar 21;120(8):1655–65. doi: 10.1111/add.70041 (PMC12215216; doi:10.1111/add.70041)
Supplement: Supplementary file 1 — Data S1. Supporting Information [file ADD-120-1655-s001.pdf]

Current and future trends in the consumption, sale and purchasing of alcohol free and low- alcohol products in Great Britain, 2014 to 2023

Luke B. Wilson, PhD[1†*]

Abigail K. Stevely, PhD [1†]

Inge Kersbergen, PhD [1]

Ellen McGrane, MSc[1]

Esther C. Moore, MSc [1]

Rob E. Pryce, PhD [1]

Prof Jamie Brown, PhD [2]

Prof John Holmes, PhD [1]

[1] Sheffield Addictions Research Group, School of Medicine and Population Health, University of Sheffield, Sheffield, S1 4DA, UK

[2] Department of Behavioural Science and Health, UCL, London, WC1E 6AE, UK

[†] Joint first authors

[*] Corresponding author: Luke B. Wilson, l.b.wilson@sheffield.ac.uk

Word Count:

Short title: Trends in no- and low alcohol

Keywords: Alcohol drinking, Zero-alcohol, Consumption, Purchasing, Time series analysis, Forecasting

1. Indicators and data sources

A1: Indicators of no/lo alcohol use and summary of datasets

Indicators	Data source	Number of time points	Granularity	Range
**Forecasts**				
1. Percentage of total alcohol sales volume that are no/lo in the on-trade	CGA	116	Monthly	June 2014 - December 2023
2. Percentage of total alcohol sales volume that are no/lo in the off-trade	Circana	208	Weekly	January 2020 - December 2023
3. Percentage of pubs and bars offering a no/lo beer on draught	CGA	124	4-weekly	June 2014 - December 2023
4. Percentage of households who do not normally purchase alcohol that are purchasing no/lo products	KWP	66	4-weekly	January 2018 - December 2023
5. Percentage of increasing risk* households who are increasing their purchasing of no/lo products (relative to alcohol products) in the off-trade	KWP	66	4-weekly	January 2018 - December 2023
6. Percentage of households who are increasing their purchasing of no/lo products and decreasing their consumption of alcohol products	KWP	66	4-weekly	January 2018 - December 2023
7. Percentage of hazardous or harmful drinkers self-reporting using no/lo products in their most recent attempt to cut down	ATS	34	Monthly	October 2020 – December 2023
**Descriptives***				
Percentage of moderate drinkers self-reporting using no/lo products in their most recent attempt to cut down	ATS	3	Every 2-4 months	Oct 2022 – Apr 2023
Percentage of respondents who do not consume alcohol products who consume no/lo products	ATS	4	Every 2-4 months	Aug 2022 – Apr 2023
**Notes:** * Individuals who consume more than 14 alcohol units a week (One unit equals 10ml or 8g of pure alcohol). ** Individuals who consume more than 35 alcohol units a week. *** These analyses are descriptive only due to insufficient data for robust time series analysis.				

2. Calculating servings of no/lo drinks continued

For several of our measures, we compare no/lo sales volume with standard alcohol sales volumes. Public health-oriented analyses of alcohol sales volumes typically convert natural volumes (i.e. volume of liquid) to pure alcohol volumes (i.e. volume of ethanol). However, this can lead to uninformative data for some analyses of no/lo drink sales as these products contain little or no alcohol. Analysing natural volumes is also problematic because differences in standard serving sizes and alcohol content between the main beverage types (e.g. beer, wine, spirits) can distort comparisons between groups or time-points. For example, Person A and B both drink 200g of alcohol per week. Person A does so by drinking 5,000 ml of 5·0% ABV beer and Person B does so by drinking 625 ml of 40·0% ABV spirits. If Person A drinks 500ml less beer a week their alcohol consumption will fall by 36g. However, if Person B drinks 500ml less spirits per week their alcohol consumption will fall by 160g. When aggregated to population-level sales data, this means changes in the natural volume of spirits sales have more impact on pure alcohol consumed per capita than equivalent changes in the natural volume of beer sales. This is unproblematic when analysing trends in sales by beverage type but analyses of trends in total sales that do not account for it may produce misleading conclusions for public health.

We address this problem by using 'servings' as a volume metric rather than natural volume. To calculate servings we divide the natural volume of sales by a standard serving size for each beverage type. We then sum together servings for all beverage types to give the total sales volume in servings. An alternative approach would be to assign a 'no/lo unit' value to no/lo sales based on the number of units of alcohol they would contain if they were standard alcoholic drinks (e.g. a pint of no/lo beer might have a value of 2·6 equivalent to 4·5% ABV beer, and 25ml of no/lo spirits might have a value of 0·9 equivalent to 37.5% ABV vodka). These no/lo unit values could then be combined to produce aggregate sales data.

The no/lo units approach is potentially more accurate than the servings approach in describing the alcohol consumption that is absent in no/lo consumption. However, the concept of no/lo units is less intuitive than that of servings and the accuracy of calculating no/lo units relies on having access to the ABV of no/lo and standard alcoholic products. This is available in our data but may not be available to other researchers conducting similar analyses. Therefore, to enable comparability with future no/lo research, we chose to adopt the servings approach for this analysis.

To select the standard serving sizes used for the on-trade, we used data from the Weights and Measures Act 1985 (https://www.gov.uk/weights-measures-and-packaging-the-law/specified-quantities) which specifies that alcoholic beverages must be sold in fixed sizes known as ‘specified quantities’. To select the standard serving sizes used for the off-trade, we calculated the median product volume by SKU for each beverage type in Circana, excluding volumes above one litre to prevent multi-packs of beer from skewing the results. Since wine and spirits are sold in bottles, for these beverage types we draw on estimates from experimental studies that suggest a self-poured glass of wine is typically around the size of a medium glass (i.e. 175ml) and a self-poured measure of spirits is typically around the size of a double serving (i.e. 50ml). Table 1 in the main manuscript provides a breakdown of our serving size assumptions for the on- and off-trade.

3. Alcohol Toolkit Study methodology

The ATS did not include questions on cutting down alcohol consumption in England in five waves (05/2022, 07/2022, 09/2022, 11/2022, 12/2022, 07/2023) but did include it in Wales and Scotland. We therefore used multiple imputation to estimate data for England in these waves to impute how many hazardous or harmful drinkers in England attempted to cut down on drinking and how many of those used no/lo drinks to cut down. As those five waves do hold data on AUDIT-C scores, we first excluded everyone who is not a hazardous or harmful drinker (AUDIT ≥ 8 or AUDIT-C ≥ 5). Then, we used multiple imputation to concurrently impute the values for the number of cut down attempts in the past year and whether respondents used no/lo drinks to cut down. Approximately 12 percent of participants had missing data on both of those variables, we therefore conducted 12 imputations. We used sociodemographic variables that are present in all waves (sex, age, region, social grade, AUDIT-C questions 1-3) to impute these values as predictors only.

4. Model Specification

Table A2: Model specifications for indicators modelled

**Indicators**	**Model Specification**
Percentage of total alcohol sales volume that are no/lo in the on-trade	ARIMA Log AR(1) SARIMA(1,0,0,12)<br>Monthly series
Percentage of total alcohol sales volume that are no/lo in the off-trade	ARIMA Seasonal difference (52) AR(1)<br>Weekly series
Percentage of pubs and bars offering a no/lo beer on draught	ARIMA (0,1,0)<br>Monthly series
Percentage of households who do not normally purchase alcohol that are purchasing no/lo products	ARIMA (0,1,0)<br>4-weekly series
Percentage of increasing risk households who are increasing their purchasing of no/lo products (relative to alcohol products) in the off-trade*	ARIMA (0,0,0) SARIMA(1,0,0,12)<br>4-weekly series
Percentage of households who are increasing their purchasing of no/lo products and decreasing their consumption of alcohol products	ARIMA AR(1) SARIMA(1,0,0,12)<br>4-weekly series
Percentage of hazardous or harmful drinkers self-reporting using no/lo products in their most recent attempt to cut down	ARIMA (0,0,0)<br>Monthly series
Percentage of hazardous drinkers self-reporting using no/lo products in their most recent attempt to cut down	ARIMA (0,0,0)<br>Monthly series
Percentage of harmful drinkers self-reporting using no/lo products in their most recent attempt to cut down	ARIMA (0,0,0)<br>Monthly series

5. Model validation

To test the validity of our forecasts, we split the available time series data into a training period and a subsequent test period, as detailed in the analysis section. The time-point where we split the available data into a training and test period varies across datasets as our forecasted indicators differ in when they begin and the number of time points each has (see Box 1). We aimed to use a test period that covers the first ~20% of the time points. However, for the indicators that utilised the CGA data, this fell during the first year of the Covid-19 pandemic. The likely disruption to series during this period means that a test forecast would not provide a good estimate of the accuracy of our ex-ante forecasts, since these do not fall during the pandemic.

To maximise the comparability of our test forecasts (ex-post) to the ex-ante forecasts, we set the series to missing during the COVID-19 period (January 2020 to July 2021). The model was then estimated using the training period prior to the pandemic, and one additional data point in August 2021. This post-pandemic data point was included so that model would be adjusted for any change in the level of the outcome series during the pandemic. The test period was therefore between September 2021 and the end of the available data for all series affected by this problem. However, the ex-post forecasts will provide a conservative estimate of ex-ante forecast accuracy due to the recent disruption associated with the Covid-19 pandemic.

In general, the MSE shows the largest fall in accuracy between the training period and test period, suggesting that the forecasts are reasonable overall but include some larger outliers when compared to the observed series. These outliers are emphasised by the MSE due to squaring the errors (Table 2). The MAPE can be interpreted as the percentage error in the forecast. Therefore, for example, we estimate that our ex-ante forecast of the percentage of total alcohol sales volume that are no/lo in the off-trade is out by 9.8% based on the accuracy of the ex-post forecast.

Table A3: Error statistics for ex post forecasts of no/lo indicators

**Model Statistics**	**Training Period**	**Test Period**
Percentage of total alcohol sales volume that are no/lo in the on-trade (CGA)		
MSE	1·02e-11	3·39e-6

MAE	0·0000603	0·0004126
MAPE	3·97	8·00
Observations (116)	93	23
Percentage of total alcohol sales volume that are no/lo in the off-trade (Circana)		
MSE	6·48e-8	0·0000643
MAE	0·0005354	0·0014144
MAPE	4·88	10·44
Observations (208)	166	42
Percentage of pubs and bars offering a no/lo beer on draught (CGA)		
MSE	2·09e-7	5·196836
MAE	0·1092946	0·4805384
MAPE	17·64	9·78
Observations (124)	99	25
Percentage of households who do not normally purchase alcohol that are purchasing no/lo products (KWP)		
MSE	5·89e-19	0·0017923
MAE	0·0025595	0·0117417
MAPE	7·105165	23·20931
Observations (66)	53	13
Percentage of increasing risk households who are increasing their purchasing of no/lo products (relative to alcohol products) in the off-trade (KWP)		
MSE	3·63e-07	0·0016918
MAE	0·009167	0·0156674

MAPE	9·112469	13·15305
Observations (66)	53	13
Percentage of households who are increasing their purchasing of no/lo products and decreasing their consumption of alcohol products (KWP)		
MSE	1·39e-07	0·0000324
MAE	0·0013849	0·0023149
MAPE	16·2115	25·3832
Observations (66)	53	13
Percentage of hazardous or harmful drinkers self-reporting using no/lo products in their most recent attempt to cut down (ATS)		
MSE	5·56e-13	214·7913
MAE	2·380384	5·371164
MAPE	8·09	13·88
Observations (40)	32	8
Percentage of hazardous drinkers self-reporting using no/lo products in their most recent attempt to cut down (ATS)		
MSE	5·94e-14	52·47974
MAE	2·88421	5·538424
MAPE	9·47	14·40
Observations (40)	32	8
Percentage of harmful drinkers self-reporting using no/lo products in their most recent attempt to cut down (ATS)		
MSE	1·03e-13	492·553
MAE	3·607599	7·8466
MAPE	13·36	20·40

Observations (40)	32	8

Notes: Mean Square Error (MSE), Mean Absolute Error (MAE), Mean Absolute Percentage Error (MAPE)

6. Additional Results from Alcohol Toolkit Study

Figure A1: Percentage of hazardous drinkers self-reporting using no/lo products in their most recent attempt to cut down

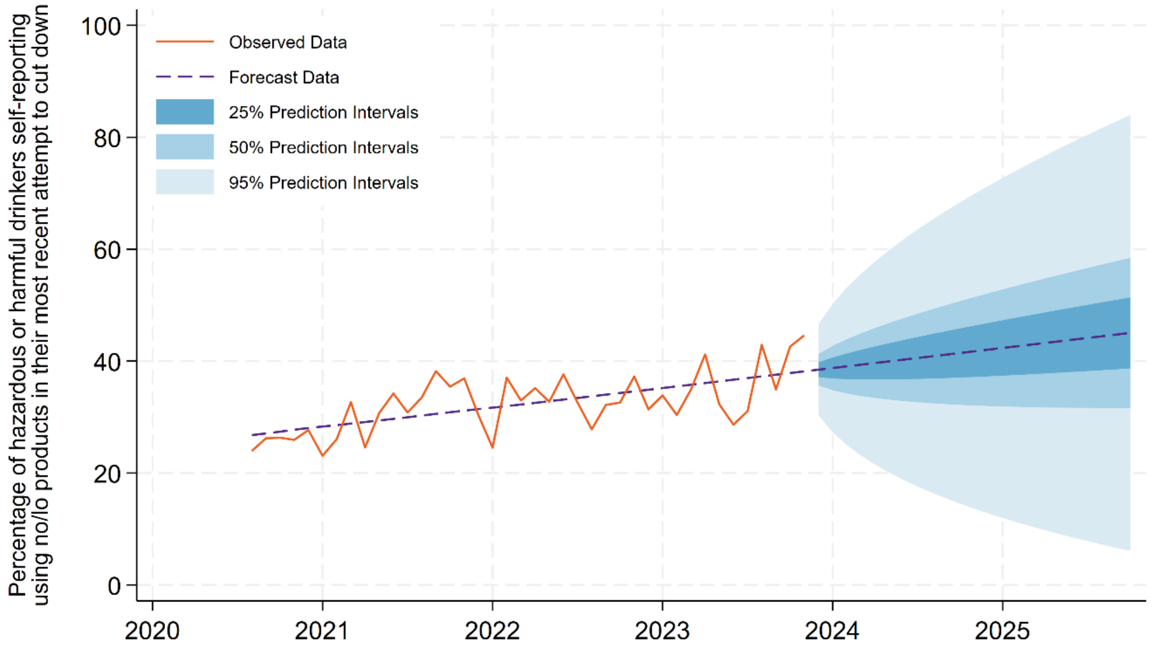

Figure A2: Percentage of harmful drinkers self-reporting using no/lo products in their most recent attempt to cut down

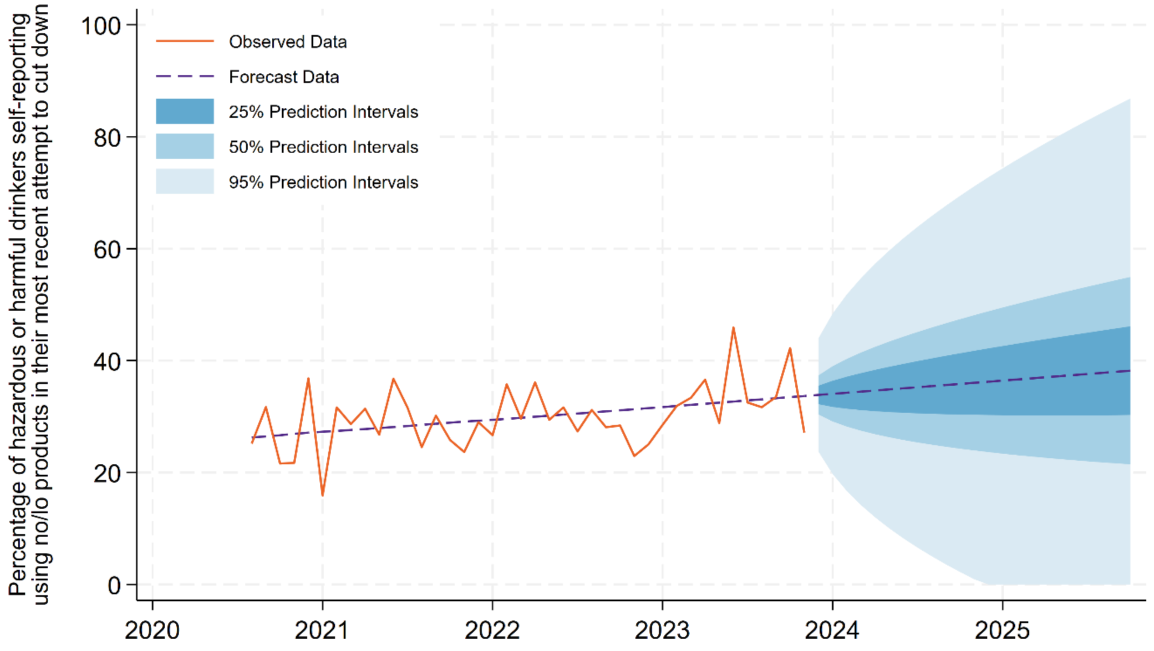

7. Model Coefficients

Table A4: Coefficients of the SARIMA models

Model Statistics	B (99% CI)	*P-value*
Percentage of total alcohol sales volume that are no/lo in the on-trade (CGA)		
Time	0·0201507<br>(0·0002527, 0·0400486)	0·009
Covid-19 (OxCGRT)	-0·0022313<br>(-0·0036984, -0·0007643)	0·000
Constant	-20·55432<br>(-36·90535, -4·203294)	0·001
AR terms	0·9206535<br>(0·8223994, 1·018908)	0·000
	0·8916415<br>(0·792447, 0·990836)	0·000
Percentage of total alcohol sales volume that are no/lo in the off-trade (Circana)		
Time	0·000016<br>(-0·0000131, 0·000045)	0·157
Covid-19 (OxCGRT)	0·000031<br>(-0·0000156, 0·000075)	0·086
Constant	-0·0505973<br>(0·1458937, 0·044699)	0·171
AR terms	L1: 0·7413319<br>(0·5964617, 0·8862021)	0·000
Percentage of pubs and bars offering a no/lo beer on draught (CGA)		
Time	5·05E-06	0·47

	(-0·0000223, 0·0000324)	
Covid-19 (OxCGRT)	3·29E-07<br>(-0·0000137, 0·0000144)	0·06
Constant	0·00006<br>(-0·0025592, 0·0026792)	0·06
AR terms	-0·09904<br>(-0·3282319, 0·1301519)	0·266
Percentage of households who do not normally purchase alcohol that are purchasing no/lo products (KWP)		
Time	2·56e-07<br>(-2·08e-06, 2·59e-06)	0·777
Covid-19 (OxCGRT)	-2·94e-06<br>(-0·0000346, 0·0000405)	0·840
Constant	-0·0053534<br>(-0·0585903, 0·0478834)	0·796
AR terms	-	-
Percentage of increasing risk households who are increasing their purchasing of no/lo products (relative to alcohol products) in the off-trade (KWP)		
Time	0·0000267<br>(0·0000149, 0·0000385)	0·000
Covid-19 (OxCGRT)	-0·0002002<br>(-0·0003293, -0·0000711)	0·000
Constant	-0·4828463<br>(-0·7535142, -0·2121784)	0·000
AR terms	0·7240223<br>(0·488923, 0·9591216)	0·000
Percentage of households who are increasing their purchasing of no/lo products and decreasing		

their consumption of alcohol products (KWP)		
Time	2·85e-06	0·089
	(-1·46e-06, 7·16e-06)	
Covid-19 (OxCGRT)	-0·0000144	0·346
	(-0·0000537, 0·0000249)	
Constant	-0·0532898	0·164
	(-0·1519185, 0·045339)	
AR terms (1)	0·492097	0·005
	(0·0401472, 0·9440468)	
AR (13)	0·5764127	0·000
	(0·3013891, 0·8514364)	
Percentage of hazardous or harmful drinkers self-reporting using no/lo products in their most recent attempt to cut down (ATS)		
Time	0·0026154	0·022
	(-0·0003312, 0·0055621)	
Covid-19 (OxCGRT)	0·0000532	0·905
	(-0·0010968, 0·0012033)	
Constant	-1·644871	0·058
	(-3·881704, 0·5919613)	
AR terms		
Percentage of hazardous drinkers self-reporting using no/lo products in their most recent attempt to cut down (ATS)		
Time	0·002985	0·055
	(-0·0010179, 0·006988)	
Covid-19 (OxCGRT)	0·0000364	0·96
	(-0·0018246, 0·0018973)	
Constant	-1·910831	0·106

	(-4·956799, 1·135137)	
AR terms	-	-
Percentage of harmful drinkers self-reporting using no/lo products in their most recent attempt to cut down (ATS)		
Time	0·0019701 (-0·0027003, 0·0066405)	0·277
Covid-19 (OxCGRT)	0·0000397 (-0·0016918, 0·0017712)	0·953
Constant	-1·176369 (-4·72401, 2·371271)	0·393
AR terms	-	-

Notes: B = coefficient, 95% Confidence Intervals (CI)

8. Descriptive Analysis

Percentage of moderate drinkers (AUDIT-C > 0 & < 5) self-reporting using no/lo products in their most recent attempt to cut down: We calculated a monthly time series for the percentage of respondents per wave who used no/lo in their most recent attempt to cut down who scored less than 5 on the AUDIT-C.

No/lo use among people who do not consume alcohol: Since August 2022, the ATS collected data on no/lo consumption in approximately quarterly waves. All respondents are asked how often they consume "an alcohol-free or low alcohol drink, that is, beer, wine, cider, spirit or other type of alcoholic drink under 1·2% ABV?". We dichotomised this variable into any no/lo consumption ("Never" vs "Once or twice a year" or more frequent) and present descriptive analysis of no/lo consumption among people who reported not consuming alcohol in the past year on the AUDIT-C.

Indicator #8: Percentage of moderate drinkers (AUDIT-C > 0 & < 5) self-reporting using no/lo products in their most recent attempt to cut down

Using data from ATS, Table A5 shows the percentage of moderate drinkers (defined as having an AUDIT-C score less than 5) who self-reported using a no/lo alcohol product in their most recent attempt to cut down their drinking. Data for this measure was first available in October 2022 where 26·0% of individuals consumed a no/lo last year. Over the next six months this has risen to 34·0%, illustrating an 8-percentage point increase in the number of moderate drinkers using no/lo as an aid to help cut down their drinking.

Table A5: Percentage of moderate drinkers (AUDIT-C > 0 & < 5) self-reporting using no/lo products in their most recent attempt to cut down

	**Did not consume any no/lo**	**Consumed no/lo in the last year**
Wave	N (%)	N (%)
Oct-22	74 (74·0%)	26 (26·0%)
Feb-23	73 (64·0%)	41 (36·0%)
Apr-23	70 (66·0%)	36 (34·0%)

Indicator #9: Percentage of respondents who do not consume alcohol products who consume no/lo products

In Table A6 we depict the percentage of respondents who do not consume alcohol products but consume no/lo products. Data on this measure was first recorded in August 2022 in the ATS. Over the four waves of recorded data our descriptive analysis shows that the percentage of respondents who do not consume alcohol products who consume no/lo products has remained consistent. This peaked at 17·1% in October 2022, however, has dropped to 12·6% as of April 2023.

Table A6: Percentage of respondents who do not consume alcohol products who consume no/lo products

	**Did not consume any no/lo**	**Consumed no/lo in the last year**
Wave	N (%)	N (%)
Aug-22	425 (86·7%)	65 (13·3%)
Oct-22	416 (82·9%)	86 (17·1%)
Feb-23	490 (86·0%)	80 (14·0%)
Apr-23	485 (87·4%)	70 (12·6%)

9. Model denominators for indicators 4, 5, 6, and 7

Table A7: Model denominators for indicators 4, 5, and 6 using KWP

Date	Indicator 4	Indicator 6	Indicator 6
31-Dec-18	1415	1580	14381
28-Jan-19	1422	1543	14365
25-Feb-19	1456	1528	14440
25-Mar-19	1465	1506	14355
22-Apr-19	1490	1504	14284
20-May-19	1529	1488	14247
17-Jun-19	1526	1479	14133
15-Jul-19	1466	1461	13876
12-Aug-19	1473	1475	14005
09-Sep-19	1487	1495	14011
07-Oct-19	1547	1466	13965
04-Nov-19	1913	1421	13944
02-Dec-19	1508	1377	13850
30-Dec-19	1499	1499	13981
27-Jan-20	1504	1481	14026
24-Feb-20	1512	1462	13962
23-Mar-20	1387	1441	13579
20-Apr-20	1378	1504	13736
18-May-20	1365	1590	13898
15-Jun-20	1369	1707	14108
13-Jul-20	1366	1755	14177
10-Aug-20	1395	1840	14523
07-Sep-20	1440	1930	14746
05-Oct-20	1495	1970	14956
02-Nov-20	1786	2026	15085
30-Nov-20	1469	2046	15153
28-Dec-20	1472	2263	15461
25-Jan-21	1469	6471	19782
22-Feb-21	1490	2305	15770
22-Mar-21	1543	2328	15913
19-Apr-21	1579	2341	16086
17-May-21	1588	2255	15936
14-Jun-21	1581	2213	15858
12-Jul-21	1553	2178	15614
09-Aug-21	1527	2126	15513
06-Sep-21	1538	2086	15462
04-Oct-21	1589	2015	15294
01-Nov-21	1889	1959	15152
29-Nov-21	1504	1858	14929
27-Dec-21	1466	1959	14940
24-Jan-22	1460	1884	14854
21-Feb-22	1469	1840	14692
21-Mar-22	1463	1765	14370
18-Apr-22	1483	1727	14186
16-May-22	1481	1672	13959

13-Jun-22	1483	1631	13729
11-Jul-22	1448	1584	13327
08-Aug-22	1430	1562	13227
05-Sep-22	1458	1538	13096
03-Oct-22	1522	1506	12961
31-Oct-22	1870	1451	12800
28-Nov-22	1492	1399	12739
26-Dec-22	1501	1513	12788
23-Jan-23	1470	1475	12728
20-Feb-23	1492	1454	12683
20-Mar-23	1486	1418	12604
17-Apr-23	1495	1402	12537
15-May-23	1505	1373	12505
12-Jun-23	1512	1384	12485
10-Jul-23	1512	1369	12366
07-Aug-23	1496	1378	12382
04-Sep-23	1510	1365	12385
02-Oct-23	1555	1331	12396
30-Oct-23	1940	1329	12435
27-Nov-23	1538	1281	12508
25-Dec-23	1559	1366	12532

Notes:
Indicator 4: *Percentage of households who do not normally purchase alcohol that are purchasing no/lo products (off-trade only).*
Indicator 5: *Percentage of increasing or higher risk households who are increasing their purchasing of no/lo products relative to alcohol products (off-trade only).*
Indicator 6: *Percentage of households who are increasing their purchasing of no/lo products and decreasing their purchasing of alcohol products.*

Table A8: Model denominators for indicator 7 using ATS

Date	Indicator 7
10/2020	213.3571
11/2020	218.6429
12/2020	176.4286
1/2021	260.2143
2/2021	180.9286
3/2021	179.8571
4/2021	200.0714
5/2021	222.2857
6/2021	220.4286
7/2021	220.2143
8/2021	185.9286
9/2021	197.5714
10/2021	190.9286
11/2021	222.5
12/2021	168.5714
1/2022	204
2/2022	182.8571
3/2022	191.6429
4/2022	199.9286
5/2022	179.5714
6/2022	187.4286
7/2022	217.5
8/2022	186.2857
9/2022	229.1429
10/2022	206.2143
11/2022	202.7857
12/2022	222
1/2023	194.5
2/2023	180.1429
3/2023	217.3571
4/2023	162.6429
5/2023	226.8571
6/2023	192.3571
7/2023	210.3571
8/2023	183.4286
9/2023	199.3571
10/2023	204.7857
11/2023	204.6429
12/2023	218.4286
1/2024	223.2143

Notes: Indicator 7 uses pooled estimates for model denominators using multiple imputation. Indicator 7: *Percentage of hazardous or harmful drinkers who are trying to cut down their alcohol consumption that used no/lo products in their most recent attempt.*
